# Treatment Effect of Bumetanide in Children With Autism Spectrum Disorder: A Systematic Review and Meta-Analysis

**DOI:** 10.3389/fpsyt.2021.751575

**Published:** 2021-11-15

**Authors:** Tiantian Wang, Ling Shan, Chunyue Miao, Zhida Xu, Feiyong Jia

**Affiliations:** ^1^Department of Developmental and Behavioral Pediatrics, The First Hospital of Jilin University, Jilin University, Changchun, China; ^2^Department of Psychiatry, GGz Centraal, Amersfoort, Netherlands

**Keywords:** autism, bumetanide, children, treatment, meta-analysis

## Abstract

**Background:** The therapeutic effect of bumetanide on autism spectrum disorder (ASD) seems to be controversial. To obtain better evidence on the efficacy of bumetanide, a systematic review and meta-analysis were performed.

**Methods:** Randomized, placebo-controlled trials (RCTs) of bumetanide treatment in children with ASD were identified through systematic review from database inception to January 17, 2021. Subsequently, a meta-analysis was carried out to examine the effect of bumetanide on the severity of symptoms of ASD as assessed by the Childhood Autism Rating Scale (CARS) and Social Responsive Scale (SRS); core symptoms according to criteria of the *Diagnostic and Statistical Manual of Mental Disorders* (DSM)-5 of the American Psychiatric Association [social affect (SA), restricted, repetitive patterns of behavior, interests, or activities (RRB) and sensory symptoms]; and the therapeutic effect as assessed by Clinical Global Impressions-Efficacy (CGI-E).

**Results:** In total, six RCTs involving 496 participants with ASD were identified in our study. The results showed that bumetanide could significantly improve the severity of the ASD symptoms measured by CARS and SRS. There was also evidence that bumetanide had positive effect on the core symptoms of ASD such as the SA and RRB, but there was no statistically significant effect on sensory symptoms. A significant positive effect on CGI-E scores in ASD patients was also observed.

**Conclusion:** Our meta-analysis provided some support that bumetanide could improve the symptoms of children with ASD. However, additional large-scale longitudinal studies that provide clearer information and better control for confounding factors are needed to confirm our findings.

## Introduction

Autism spectrum disorder (ASD) is a neurodevelopmental disorder that affects an estimated 2% of American children (1), presenting with both abnormal communication and behavioral interactions as well as restricted repetitive sensorimotor behaviors as defined by the *Diagnostic and Statistical Manual of Mental Disorders* (DSM)-5 published by the American Psychiatric Association (2, 3). Educational and behavioral interventions, such as Joint Attention Symbolic Play, Engagement Regulation (JASPER), Early Start Denver Model (ESDM), Pivotal Response Treatment (PRT), and Early Social Interaction (ESI) are mainstays of ASD treatment (2). The availability of interventional resources is highly variable between regions and countries, however, and pharmacological intervention represents a promising alternative, particularly in cases when behavioral interventions are not available. No drugs have been approved by the Federal Drug Administration or the European Medicine Agency to treat core symptoms of ASD. Risperidone and aripiprazole have been reported to be effective in improving irritability or agitation; however, these drugs have undesirable side effects (4, 5).

Several studies have shown that bumetanide may provide a new therapeutic strategy (6, 7). According to research results, dysfunctional GABAergic (γ-aminobutyric acid) transmission and imbalanced excitatory inhibitory (E-I) neurotransmission in the brain may be related to autism (8, 9). Intracellular neuronal chloride (Cl) concentrations determine the efficacy of GABAergic inhibition (10). In both animal ASD models and ASD patients, GABAergic signaling, and Cl levels have been found to be altered, much as they are in certain other developmental disorders (11–13). Bumetanide can lower Cl levels and shift GABA from excitation toward inhibition, making it a promising tool for treating ASD (14). Bruining et al. (15) reported the paradoxical actions of benzodiazepines in a patient in which bumetanide was effective. The same group has shown that bumetanide attenuates the autistic features and symptoms in patients with tuberous sclerosis without altering the epilepsy components. These findings also illustrate the relevance of the underlying mechanisms (16).

Lemonnier and Ben-Ari (7) evaluated the effect of 3 months of bumetanide (1 mg/d) treatment on five children with ASD. The result showed that bumetanide improved autism symptoms without any side effects. This was the first study to use bumetanide to treat children with autism. To further explore the therapeutic effect of bumetanide, Lemonnier et al. (17) conducted a randomized controlled trial (RCT) with 60 children with autism or Asperger syndrome receiving placebo or bumetanide (1 mg/d for 3 months). Compared with the placebo group, symptoms in children in the bumetanide group monitored by the Childhood Autism Rating Scale (CARS) and Clinical Global Impressions (CGI) were significantly improved (17). Occasional mild hypokalemia was the only notable side effect. Thereafter, Lemonnier et al. (18) further assessed dosage/response and safety/effectiveness of bumetanide in a multicenter study. Their results revealed that bumetanide improved the core symptoms of ASD and had a promising benefit/risk ratio, especially when administered twice per day at a dose of 1.0 mg. Positive results of bumetanide in children with autism have also been reported in one study from Sweden and two studies of the same team from China (19–21). Moreover, the two Chinese studies tried to assess possible neuropharmacological mechanisms of bumetanide in young children with ASD by magnetic resonance spectroscopy (MRS). One study demonstrated that the improvement after bumetanide treatment was related to reduced GABA/Glx (glutamate) ratios in both the insular cortex (IC) and visual cortex (VC) (21). The other study found bumetanide reduced the level of GABA in the IC, which was related to the reduction of symptoms (19). Hadjikhani et al. (22) performed an open-label trial pilot study in which they used functional magnetic resonance imaging (MRI) and neuropsychological tests to determine the effect of 10 months of bumetanide treatment in adolescents and young adults with autism. They found that bumetanide was able to enhance the recognition of emotions and the activation of brain regions associated with emotional and social perceptions when processing faces displaying emotion. Subsequently, Hadjikhani et al. (23) completed their aforementioned study and found that bumetanide treatment in patients with autism could normalize the activation of the amygdala in response to eye contact and increase the spontaneous gaze time when viewing dynamic emotional faces. Regarding prolonged treatment, Grandgeorge et al. (24) used bumetanide in a 10-year-old girl diagnosed with Asperger syndrome, using Dunn's Sensory Profile to evaluate sensory behaviors. Following an 18-month course of bumetanide, this girl exhibited improvements in multisensory, auditory, tactile, vestibular, and oral sensory processing.

In contrast to the above trials, there are also studies that do not support the effectiveness of bumetanide in autism. Sprengers et al. (25) conducted an RCT of bumetanide in children with ASD, and their results indicated that bumetanide was no better than the placebo with respect to the primary outcome (Social Responsive Scale, SRS); however, it was observed that bumetanide had a significant effect on the secondary outcome of repetitive behaviors. No significant effects on other scales in terms of the Sensory Profile (SP-NL) or irritable behavior as the secondary outcomes were observed. The study of Du et al. (26) employed intensive behavioral therapy for treating all subjects, and there was no significant difference in CARS scores between the bumetanide and control groups.

Inconsistencies in results make it challenging for the clinicians to draw a definite conclusion about the efficacy of bumetanide in ASD. To improve this situation, it is important to review all current evidence-based trials to assess the risks and benefits associated with bumetanide in the treatment of ASD. The purpose of this study was to conduct a meta-analysis of RCTs to measure the effectiveness of bumetanide in children with ASD.

## Methods

The present analysis was conducted according to PRISMA guidelines.

### Selection Criteria

#### Type of Studies

RCTs were included.

#### Type of Participants

Study subjects were children <18 years of age diagnosed with ASD according to the criteria of the DSM-IV (autism, Asperger syndrome, or PDD-NOS), DSM-5, or *International Classification of Diseases-10* (ICD-10). Diagnosis was confirmed with the Autism Diagnostic Interview–Revised (ADI-R) and/or Autism Diagnostic Observation Schedule (ADOS).

#### Type of Interventions

Studies focused on assessing bumetanide safety and efficacy in children with ASD.

#### Type of Outcome

The studies included at least one outcome measurement of the core symptoms of ASD, according to the Diagnostic Criteria of DSM-5 [A, social interaction and social communication; B, restricted, repetitive patterns of behavior, interests, or activities (RRB) and sensory symptoms]. In our study, social interaction and social communication are collectively referred to as social affect (SA). We gave priority to measurements assessing the severity of the global [CARS, SRS, ADOS, Autism Behavior Checklist (ABC), and CGI] and the core (SA, RRB, and sensory issues) symptoms of ASD as the primary outcome. We included other outcomes (irritability, MRS) that can also reflect the treatment effect as secondary outcomes.

### Electronic Search

Two authors examined the following electronic databases: PubMed, Web of Science, Embase, Cochrane Library, and Dayi100 databases from inception through January 17, 2021, using the following search terms: (“autism spectrum disorder,” OR “autistic,” OR “autism,” OR “Kanner^*^ Syndrome,” OR “ASD”) AND (“bumetanide” OR “Bumex” OR “Drenural” OR “Miccil” OR “Bumedyl” OR “Burinex”). No restrictions or database filters were applied for language, time period, or year of publication. After removing duplicates, both authors reviewed all article titles and abstracts. They also independently conducted a full text review based on selection criteria. Discrepancy was solved by reaching consensus between the two authors and discussion with a third reviewer was employed if necessary.

### Data Extraction, Risk of Bias Assessment, and Grades of Recommendation, Assessment, Development, and Evaluation (GRADE) Evidence

Two authors used specially designed data extraction tables to independently extract data from selected trials. Disagreements were resolved through discussions with another senior author. The following information was independently extracted from the included trials by the two authors: the last name of the first author, publication year, country of origin, total sample size, age of children, dosage and duration of bumetanide supplementation, the intervention in the control group, outcome measurement, and adverse effects.

The authors employed the Cochrane Collaboration Risk Assessment tool to assess the risk of bias for included studies based upon allocation concealment, random sequence generation, blinding of participants and investigators, blinding of outcome assessment, complete outcome data, and selective reporting of outcome or biases from additional sources.

The GRADE system was used to evaluate the power of recommendation and the quality of the evidence. The GRADEpro software program was used to construct the tables. We used a four-point scale (“high,” “moderate,” “low,” or “very low”) to indicate the quality of the evidence (Table 1).

**Table 1:** GRADE table for outcomes included in the meta-analysis.

**Outcome**	**Quality assessment**							**No of patients**		**Effect**		**Quality**
	**No. of studies**	**Design**	**Risk of bias**	**Inconsistency**	**Indirectness**	**Imprecision**	**Other considerations**	**Bumetanide**	**Control**	**Relative (95% CI)**	**Absolute**	
CARS	5	RCTs	Serious^1^	Not serious	Not serious	Not serious	None	222	177	–	MD 1.35 lower (2.06–0.64 lower)	⊕⊕⊕◯ MODERATE
SA	10	RCTs	Serious^2^	Not serious	Not serious	Not serious	None	423	351	–	SMD 0.46 lower (0.61–0.31 lower)	⊕⊕⊕◯ MODERATE
RRB	9	RCTs	Serious^2^	Not serious	Not serious	Not serious	None	391	361	–	SMD 0.24 lower (0.39–0.1 lower)	⊕⊕⊕◯ MODERATE
Sensory	2	RCTs	Serious^2^	Not serious	Not serious	Serious^b^	None	66	62	–	SMD 0.16 lower (0.51 lower to 0.19 higher)	⊕⊕◯◯ LOW
SRS	3	RCTs	Not serious	Not serious	Not serious	Serious^2^	None	114	88	–	MD 8.77 lower (15.59–1.95 lower)	⊕⊕⊕◯ MODERATE
CGI-E	2	RCTs	Serious^2^	Not serious	Not serious	Not serious	None	56	53	–	MD 0.27 higher (0.09–0.44 higher)	⊕⊕⊕◯ MODERATE

*RCTs, randomized controlled trials; CI, Confidence interval; MD, weighted mean difference; SMD, standardized mean difference; CARS, Childhood Autism Rating Scale; SA, social affect; RRB, restricted, repetitive patterns of behavior, interests, or activities; SRS, Social Responsive Scale; CGI-E, Clinical Global Impressions–efficacy*.

1,2 *Means the number risk of bias of each study*.

### Data Analysis

Review Manager v5.2 was applied to the current analysis. The mean and standard deviation were used to compare continuous data. When two or more studies were satisfactory and met inclusion criteria, a meta-analysis of their findings was performed. Some studies reported scores measured at 3 months, while other studies only showed the changes in scores from baseline. Therefore, we analyzed them together. Sometimes there were several treatment groups in one study based on the dosage of bumetanide. The data of these bumetanide groups were integrated into one treatment group. Effect size of differences between the control and bumetanide treatment groups was compared by weighted mean difference (WMD) if the studies measured the results in a uniform way. Otherwise, standardized mean difference (SMD) was used. When *I*^2^ was 50% or below, a fixed-effects model was applied to calculate these WMD or SMD values and the corresponding 95% confidence intervals (CIs) by the Mantel–Haenszel approach. Because of the heterogeneity, data were compared with random effects models through the DerSimonian and Laird methods. No subgroup analysis or meta-regression was performed because of the limited number of studies in the present meta-analysis.

## Results

**Figure 1:**
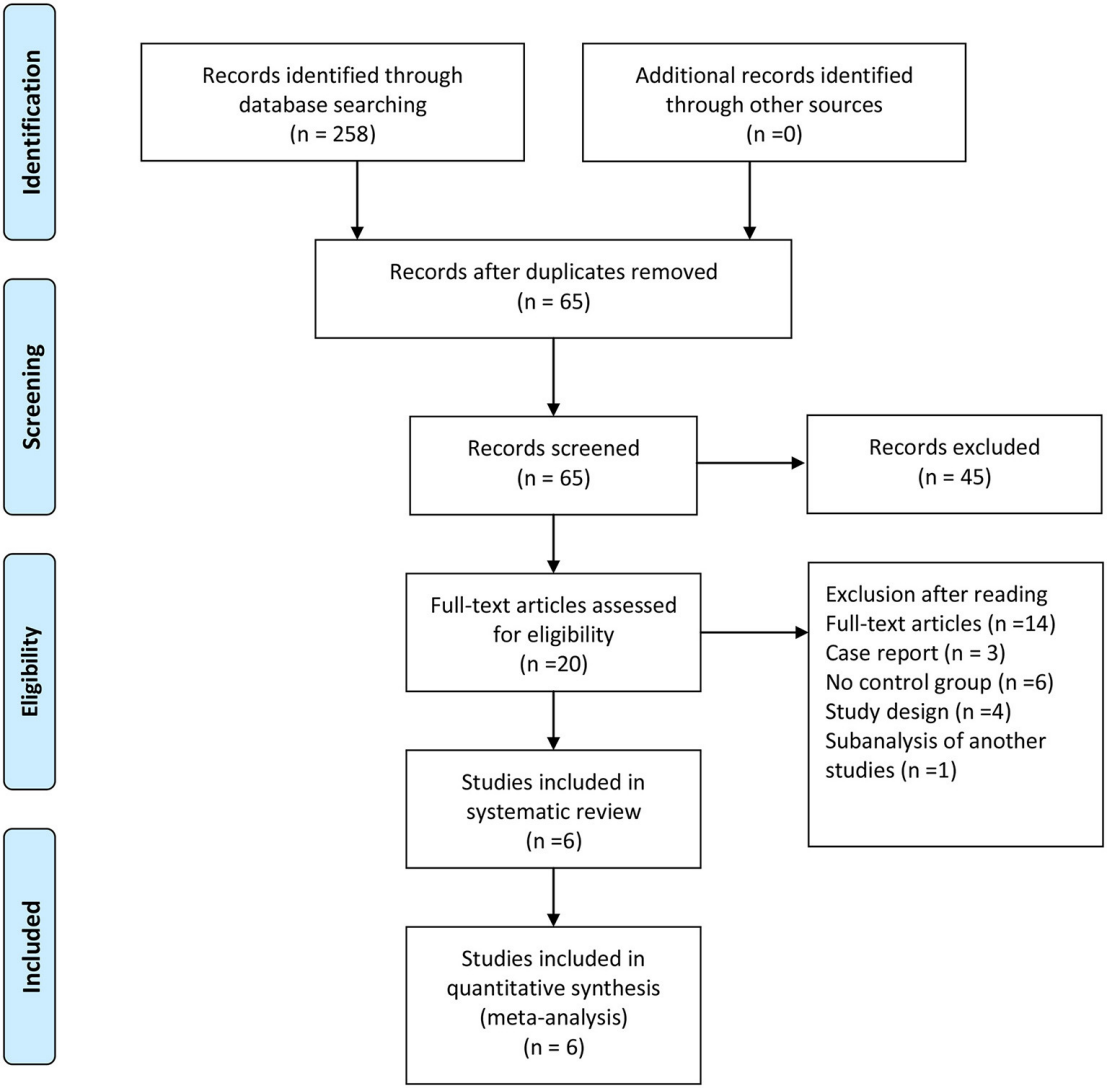
Flow diagram for selection of studies (PRISMA flow diagram).

In total, 258 studies were found in our preliminary search, of which 193 were duplicates. After removing duplicates, 65 articles were excluded by filtering abstracts or titles, and only 20 articles were evaluated for eligibility. After reading the full text, only six studies met the selection criteria for inclusion in the final analysis. The reasons for excluding the 14 full-text articles were as follows: four studies were excluded, because they are study designs with no results; three studies were case reports, six studies were missing a control group, and one study was a sub-analysis of another study. The screening and selection of the studies are presented in the PRISMA flowchart, shown in Figure 1. Table 2 displays the sample characteristics of the six included studies.

**Table 2:** Characteristics of the RCTs included in the systematic review.

**Study**	**Country**	**Number**	**Age**	**Intervention**		**Duration**	**Outcomes**	**Adverse effects**	
			**(year)**	**Bumetanide**	**Control**	**(month)**		**Bumetanide**	**Control**
Lemonnier et al. (17)	France	54 (27/27)	3–11	0.5 mg bid	Placebo	3	CARS, CGI, ADOS	Bed-wetting, hypokalemia	Bed-wetting, eczema
Du et al. (26)	China	60 (28/32)	2.5–6.5	0.5 mg bid +ABA	ABA	3	ABC, CARS, CGI	Polyuria	–
Lemonnier et al. (18)	France	88 (65/23)	2–18	0.5 mg, 1.0 mg, 2.0 mg bid	Placebo	3	CARS, SRS, CGI	Hypokalemia, polyuria, anorexia, diarrhea	Diarrhea,
Sprengers et al. (25)	Netherlands	92 (47/45)	7–15	1.0 mg bid	Placebo	3	SRS, RBS-R, SP-NL, Aberrant Behavior Checklist Irritability Subscale	Orthostatic hypotension, hypokalemia, hypoglycemia	orthostatic hypotension, dehydration, hypoglycemia
Zhang et al. (21)	China	83 (42/41)	3–6	0.5 mg bid	–	3	CARS, CGI, GABA/NAA, GABA/Glx	Hypokalemia, polyuria, loss of appetite, fatigue, hyperuricemia	–
Dai et al. (19)	China	119 (59/60)	3–6	0.5 mg bid	Placebo	3	CARS, SRS, ADOS, CGI, MRS	Polyuria, hypokalemia, nausea, hyperuricemia, loss of appetite, constipation, vomiting, sleeping problem	Polyuria, hyperuricemia, vomiting, loss of appetite, constipation, diarrhea

*CARS, Childhood Autism Rating Scale; CGI, Clinical Global Impressions; AODS, Autism Diagnostic Observation Schedule; ABC, Autism Behavior Checklist; SRS, Social Responsive Scale; RBS-R, Repetitive Behavior Scale-Revised; SP-NL, Sensory Profile; Glx, glutamate; GABA, γ-aminobutyric acid; NAA, n-acetylaspartate; MRS, magnetic resonance spectroscopy*.

### Risk of Bias

**Figure 2:**
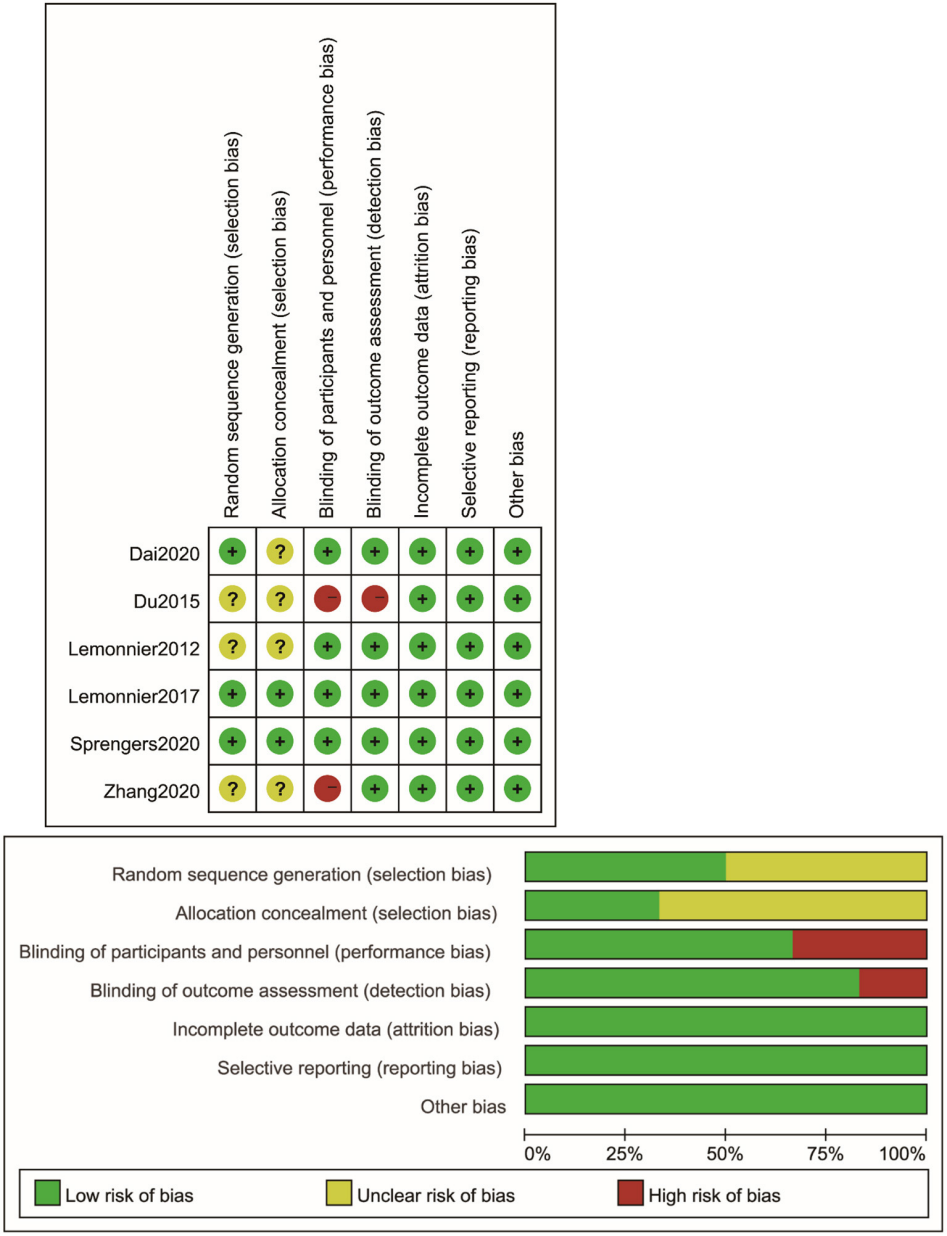
Risk of bias in individual and across studies.

See Figure 2 for the results on the risk of bias of these included studies. Three studies (18, 19, 25) clearly used the method of random sequence generation and were considered to be low risk. Three studies (17, 21, 26) failed to explain their random sequence generation and were rated as unclear risk. Two studies (18, 25) used allocation concealment techniques and were considered to be low risk. The remaining studies (17, 19, 21, 26) did not mention the details of allocation concealment and exhibited an unclear risk of bias. For blinding in terms of performance bias, two studies (21, 26) did not blind with respect to participants and were considered to be high risk. The other four studies (17–19, 25) had a clear blinding design and were classified as low risk. For blinding in terms of detection bias, four studies were clearly considered to be low risk, and one study was deemed high risk because it was not blinded to the raters. All articles were considered to be low risk for incomplete outcome data (attrition bias) and selective reporting (reporting bias).

### The Severity of ASD Symptoms

#### CARS

**Figure 3:**
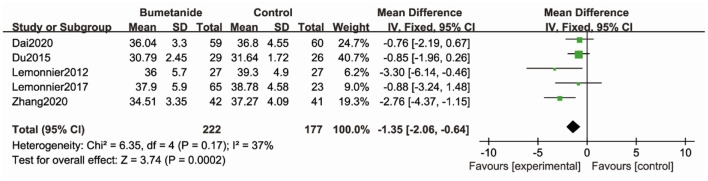
Forest plot of the efficacy of bumetanide on CARS in children with ASD.

Five studies (17–19, 21, 26) used CARS to assess the severity of symptoms of ASD. CARS, a behavioral rating scale, is used for assessing the severity of the symptoms of ASD. CARS evaluates emotional and sensory response, communication, adaptative ability, and activity level. The general efficacy of the bumetanide treatment was significantly superior relative to the control group [WMD 95% CI = −1.35 (−2.06, −0.64), *p* = 0.0002] by comparing the total CARS score. The studies included in this analysis did not show significant heterogeneity (*I*^2^ = 37%) (Figure 3).

#### SRS

**Figure 4:**
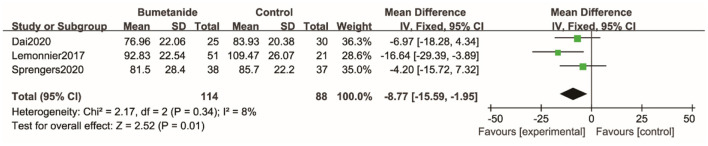
Forest plot of the efficacy of bumetanide on SRS in children with ASD.

Three studies (18, 19, 25) used SRS to assess the severity of symptoms of ASD. SRS evaluates the following areas: cognition, social awareness, motivation, communication, restricted interests, and repetitive behavior. The general efficacy of the bumetanide was significantly better than that of the control group [WMD 95% CI = −8.77 (−15.59, −1.95), *p* = 0.01] by comparing the total score of SRS. The studies included in this analysis did not show significant heterogeneity (*I*^2^ = 8%) (Figure 4).

#### ADOS

Two studies (17, 19) used ADOS to assess severity of autism. ADOS is a semi-structured observational tool that can evaluate social interaction, communication, imagination, and play ability. In Dai et al. (19), the total scores of ADOS were missing; therefore, no results from this study could be incorporated into the present meta-analysis. The other study found efficacy of bumetanide was not significantly better than in the control group (17).

#### Autism Behavior Checklist (ABC)

The Du et al. study (2015) was the only one that assessed the effect of bumetanide on ASD symptoms using the ABC. The bumetanide group showed a statistically significant better treatment effect in terms of ABC scores (*p* < 0.05) (26).

### CGI

**Figure 5:**
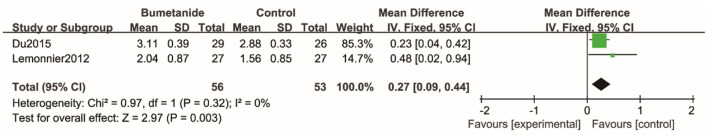
Forest plot of the efficacy of bumetanide on CGI-E in children with ASD.

Three studies used the Clinical Global Impressions efficacy index (CGI-E) to evaluate clinical efficacy on global symptoms and associated side effects (17, 21, 26). The scores in one study (21) were missing. The global effect of the bumetanide was significantly superior to the control group [WMD 95% CI = 0.27 (0.09, 0.44), *p* = 0.003]. The studies included in the analysis did not show significant heterogeneity (*I*^2^ = 0%). Four studies (17, 18, 21, 26) evaluated degree of improvement in the global symptoms relative to the baseline using Clinical Global Impressions–Improvement scale (CGI-I). Because of the lack of complete data, the study results could not be included in the meta-analysis. All four studies found that improvement of symptoms relative to the baseline in the bumetanide group was significantly better than in the control group. Du et al. (26) was the only study to evaluate the effect of bumetanide with Clinical Global Impressions severity of illness (CGI-S) and showed that the bumetanide group had a better effect on the severity of global symptoms compared with the control group (*p* < 0.05) (26) (Figure 5).

### SA

**Figure 6:**
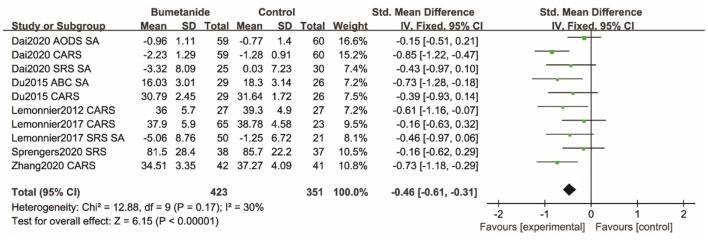
Forest plot of the efficacy of bumetanide on SA in children with ASD. ADOS, Autism Diagnostic Observation Schedule; CARS, Childhood Autism Rating Scale; SA, Social Affect; SRS, Social Responsive Scale; ABC, Autism Behavior Checklist.

SRS, SRS social interaction sub-domains, ADOS social and communication sub-domain, ABC interaction sub-domain, and CARS were used to assess social interaction and social communication (SA). Figure 6 shows the forest plot of the 10 SA results reported by the six studies (17–19, 21, 25, 26). The global efficacy of bumetanide was significantly better than that of the control group [SMD 95% CI = −0.46 (−0.61, −0.31), *p* < 0.00001]. The studies included in the analysis did not show significant heterogeneity (*I*^2^ = 30%).

### RRB

**Figure 7:**
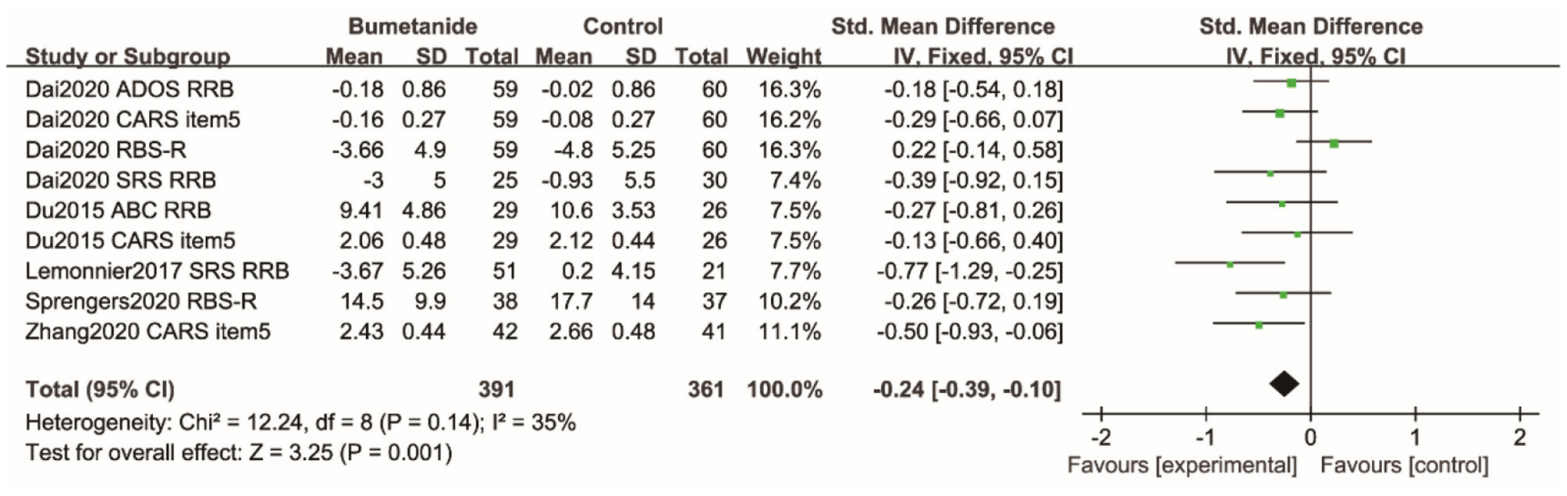
Forest plot of the efficacy of bumetanide on RRB in children with ASD. ADOS, Autism Diagnostic Observation Schedule; RRB, Restricted, repetitive patterns of behavior, interests, or activities; CARS, Childhood Autism Rating Scale; RBS-R, Repetitive Behavior Scale-Revised; SRS, Social Responsive Scale; ABC, Autism Behavior Checklist.

Repetitive Behavior Scale-Revised (RBS-R), RRB sub-domain of the SRS, RRB sub-domain of the AODS, RRB sub-domain of the ABC, and CARS item 5 were used to assess RRB. Figure 7 shows the forest plot of the nine RRB results reported by five studies (18, 19, 21, 25, 26). The global efficacy of bumetanide was significantly superior to the control group [WMD 95% CI = −0.24 (−0.39, −0.10), *p* = 0.001]. The studies included in the analysis did not show significant heterogeneity (*I*^2^ = 35%).

### Sensory Issues

**Figure 8:**
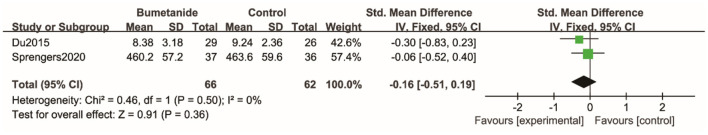
Forest plot of the efficacy of bumetanide on Sensory in children with ASD.

Two studies (25, 26) used SP-NL and the ABC sensory sub-domain to evaluate sensory scores between the bumetanide and the control groups, but there were no statistical differences between the two groups [SMD 95% CI = −0.16 (−0.51, 0.19), *p* = 0.36]. Studies incorporated into this analysis did not show significant heterogeneity (*I*^2^ = 0%) (Figure 8).

### Irritability

Sprengers et al. (25) was the only study that used the Aberrant Behavior Checklist to assess the effect of bumetanide on irritability and found no significant difference between bumetanide and the control group.

### MRS

There were two studies that measured concentration of neurotransmitters by MRS (19, 21). Zhang et al. (21) reported that the GABA/Glx ratio in the IC and VC in bumetanide group decreased faster than in the control group within 3 months. The decrease in the IC was related to the symptom improvements in the bumetanide group (21). Similarly, Dai et al. (19) found that bumetanide had an advantage over placebo in reducing GABA concentration in the IC and the clinical improvement was related to decreased GABA concentration in the IC in the bumetanide group.

### Adverse Events

We were not able to conduct a meta-analysis of adverse events because of insufficient data. In the study of Dai et al. (19), adverse events were mild and did not require discontinuation of the drug. The adverse events observed were as follows: polyuria (67.8%), hypokalemia (8.5%), mild hyperuricemia (3.4%), loss of appetite (6.8%), constipation (8.5%), nausea (8.5%), vomiting (1.7%), and sleeping problems (1.7%) (19). Du et al. (26) reported that one participant discontinued medication because of polyuria. However, the study did not describe the severity of polyuria. In the Lemonnier et al. (17) study, two participants terminated the research because of bed-wetting and hypokalemia, respectively. Six children treated with bumetanide had mild hypokalemia and were supplemented with potassium-gluconate syrup. In the Lemonnier et al. (18) study, the most frequent treatment-emergent adverse events (TEAEs) included hypokalemia, diuresis and loss of appetite, dehydration, and asthenia. The frequency and incidence of adverse events were directly correlated with the dosage of bumetanide, with a favorable benefit/risk ratio especially at 1.0 mg twice daily. Sprengers et al. (25) reported that hypokalemia, orthostatic hypotension, dehydration, and diuresis were the most common and frequent expected adverse events. All events were mild to moderate in intensity and were resolved. Zhang et al. (21) reported that the most common adverse effects observed were mild polyuria/pollakiuria, mild hypokalemia, loss of appetite, fatigue, and mild hyperuricemia. They required no additional treatment or were resolved.

## Discussion

The purpose of this meta-analysis is to evaluate the efficacy and safety of bumetanide in the treatment of children with ASD. We analyzed six RCTs consisting of 496 children to test whether bumetanide can improve ASD symptoms as assessed by validated outcome indicators. Not only symptomatic improvement in terms of severity of symptoms of ASD but the core domains (SA and RRB) of ASD described in the DSM-5 were also examined. These analyses revealed that the bumetanide group demonstrated a significantly better effect in reducing the severity of ASD as measured by CARS and SRS relative to controls. For clinical efficacy referring to the CGI, the bumetanide group showed significantly better effect compared with the control group. There was mild to moderate heterogeneity among studies. The bumetanide group had also significantly better therapeutic effects with respect to SA and RRB compared with the control group. Furthermore, this study did not suggest that bumetanide had a significantly better effect on sensory problems.

To our knowledge, this is the first meta-analysis to evaluate the treatment effect of bumetanide in children with ASD. There was one previous meta-analysis examining the efficacy of pharmacological treatments for RRBs in ASD. Bumetanide was one of the included pharmacological options in their study and was significantly associated with the improvement of RRB outcomes compared with placebo (27). Our results are congruent with this study. In addition, more trials were included in our meta-analysis to evaluate RRB symptoms, and the SA and sensory problems of ASD were also examined. Previous studies mainly explored the effect of bumetanide on the core symptoms of ASD and were mostly qualitative review studies. Mollajani et al. (28) suggested there was evidence that bumetanide could be a novel pharmacological treatment for core symptoms of ASD. James et al. (29) reviewed the role of bumetanide in the treatment of ASD and found that bumetanide could improve the total scores on a variety of autism assessment scales. They concluded that bumetanide might be useful for patients with moderate to severe ASD. Our current quantitative analysis confirmed their speculation. Regarding the evaluation method pre- and post-bumetanide treatment, MRS was also used in some included RCTs in addition to scales. These studies showed a decreased GABA concentration in the IC after bumetanide treatment in children with ASD (19, 21). Unfortunately, a quantitative analysis concerning GABA concentration could not be carried out because of the large heterogeneity. However, the two studies have shown that symptom improvement after bumetanide treatment was significantly related to the reduced GABA concentration in the IC. One included study by Zhang et al. (21) detected a reduced GABA/Glx ratio in the IC on MRS. A previous report showed the ability of bumetanide to suppress amygdala activation in adolescent ASD patients, and the authors speculated that bumetanide could restore the E-I balance in the brain (23). Another study reported elevated GABA/Glx ratios in cortical and striatal areas in a type I neurofibromatosis mouse model of autism (30). The studies included in our meta-analysis supported the conclusion that bumetanide is able to normalize E-I balance in the brains of children with ASD to promote normal brain function and social-emotional cognition. However, both the above studies were completed by the same team and the number of studies is too small to draw a firm conclusion. However, they could be preliminary evidence that bumetanide might decrease GABA concentration in the IC in children with ASD, but it does not have strong evidence-based efficacy. MRS may be a promising tool for a precision medical approach for the treatment of ASD. Current findings suggest that bumetanide is a safe and tolerated treatment in children with ASD.

### Limitations

Our meta-analysis also has limitations. First, the sample size in our meta-analysis was not large. More RCTs are needed in the future. Second, the treatment period was very short (3 months) in the trials that we included. An open pilot study of seven adolescents and young adults with autism receiving bumetanide treatment for 10 months showed that facial emotion recognition and the activation of associated brain regions were improved. Thus, the potential long-term effect of bumetanide remains to be elucidated. Third, the outcomes were assessed by various measurement tools. A wide variety of evaluation methods with different scoring systems were used in the included studies, which may not be directly comparable with each other or there may be potentially more risk for heterogeneity. Future studies will require the use of standardized measurement tools in pharmacological trials in order to aggregate smaller studies into meaningful summaries. Fourth, because of the small number of the included studies, we did not perform analysis to examine the dosage effect or the safety and tolerability of bumetanide in the ASD population.

## Conclusion

In the current meta-analysis, it was found that bumetanide has a small but significant benefit for ASD symptoms. Bumetanide is also safe and well-tolerated. In addition, we also found that GABA concentration may decrease after bumetanide treatment in children with ASD examined by MRS, which may be meaningful in clarifying the treatment mechanism of bumetanide. In the future, MRS could be widely used in the precision medical approach for the treatment of ASD. Moreover, it is still necessary to conduct more trials with larger sample sizes, better control of confounding factors, long-term follow up, and more specific details.

## Data Availability Statement

The raw data supporting the conclusions of this article will be made available by the authors, without undue reservation.

## Author Contributions

FJ and TW conceptualized the study and wrote the discussion after revisions were made. TW and LS examined the electronic databases and collected the data. Discrepancy was solved by discussion with CM if necessary. TW and CM analyzed the data and wrote the methods and results. ZX read the paper and provided suggestions for revisions. All authors read and revised the manuscript several times.

## Funding

This study was supported by the National Nature Science Foundation of China (81973054) and Key Scientific and Technological Projects of Guangdong Province (2018B030335001).

## Conflict of Interest

The authors declare that the research was conducted in the absence of any commercial or financial relationships that could be construed as a potential conflict of interest.

## Publisher's Note

All claims expressed in this article are solely those of the authors and do not necessarily represent those of their affiliated organizations, or those of the publisher, the editors and the reviewers. Any product that may be evaluated in this article, or claim that may be made by its manufacturer, is not guaranteed or endorsed by the publisher.
